# Supplementary figures and images for: High-mobility group box 1 fragment suppresses adverse post-infarction remodeling by recruiting PDGFRα-positive bone marrow cells
Source: PLoS One. 2020 Apr 10;15(4):e0230392. doi: 10.1371/journal.pone.0230392 (PMC7147742; doi:10.1371/journal.pone.0230392)

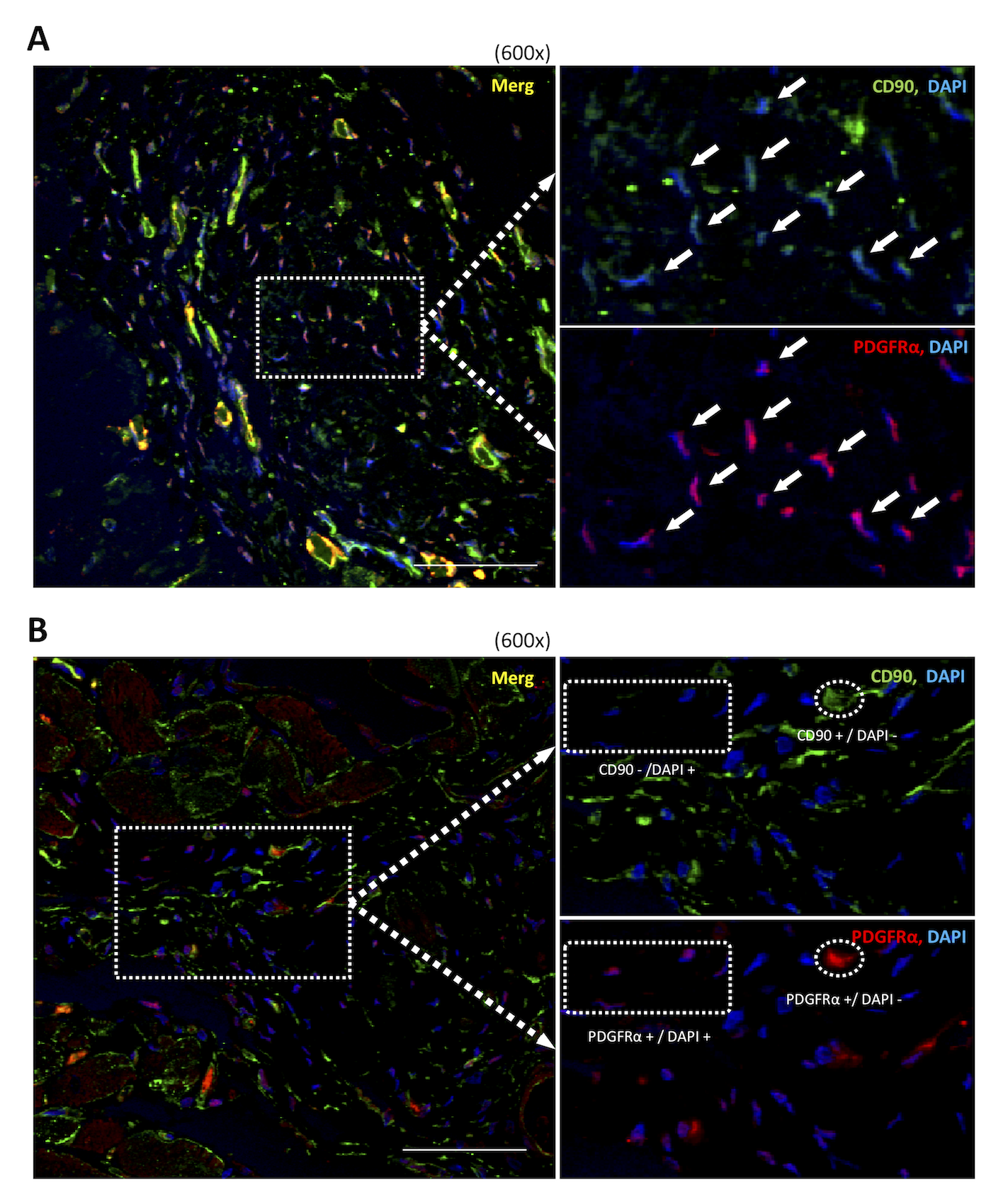

Supplement: S1 Fig — CD90+/PDGFRα+ and DAPI+ cells were counted as MSCs (A: 600×, scale bar = 50 μm, white arrows). Cells negative for CD90, PDGFRα, or DAPI were excluded (B: 600×, scale bar = 50 μm). (TIFF) [file pone.0230392.s002.tiff]
