# Supplementary material for: High-mobility group box 1 fragment suppresses adverse post-infarction remodeling by recruiting PDGFRα-positive bone marrow cells
Source: PLoS One. 2020 Apr 10;15(4):e0230392. doi: 10.1371/journal.pone.0230392 (PMC7147742; doi:10.1371/journal.pone.0230392)
Supplement: S1 File — (DOCX) [file pone.0230392.s003.docx]

**Supporting information: All data of statistical analysis in the present study**

**Examination 1**

Echocardiogram (Figure 2B)

・LVEF: HMGB1 group vs. control; pre 42.25 ± 5.07% vs. 43.94 ± 4.89%, 1w later 45.61 ± 5.926% vs. 39.15 ± 4.908%, *P* = 0.0056, 4w later 48.61 ± 5.51% vs. 33.93 ± 5.27%, *P* < 0.0001.

・LVDd: pre 0.968 ± 0.105 vs. 1.013 ± 0.086 mm, *P* = 0.24, 1w later 1.001 ± 0.082 vs. 1.076 ± 0.114 mm, *P* = 0.073, 4w later 1.064 ± 0.089 vs. 1.122 ± 0.087 mm, *P* = 0.11.

・LVDs: pre 0.792 ± 0.103 vs. 0.820 ± 0.087 mm, *P* = 0.45, 1w later 0.803 ± 0.091 vs. 0.896 ± 0.110 mm, *P* = 0.027, 4w later 0.833 ± 0.0905 vs. 0.963 ± 0.095 mm, *P*=0.0016.

Histological analysis (Figure 2C, 2D, 2E, 2F)

・Fibrotic area: 11.58 ± 5.18% vs. 23.07 ± 6.32%, *P* < 0.0001 (Figure 2C)

・Cardiomyocyte size: 19.11 ± 2.59 vs. 26.82 ± 1.36 μm, *P* < 0.0001 (Figure 2D)

・Capillary density: 1797.98 ± 271.85 vs. 959.04 ± 143.40/mm^2^, *P* < 0.0001 (Figure 2E)

・CD90^+^/PDFGRα^+^ cells in the peri-infarction area: 1636.84 ± 538.378 vs. 934.00 ± 250.236/mm^2^, *P* = 0.0003 (Figure 2F)

RT-PCR analysis (Figure 2G)

・VEGF-A mRNA: peri-infarction area 1.63 ± 0.64 vs. 1.18 ± 0.25, *P* = 0.029, septum 1.14 ± 0.11 vs. 0.99 ± 0.13, *P* = 0.0040.

・TGFβ mRNA: peri-infarction area 1.13 ± 0.25 vs. 1.66 ± 0.75, *P* = 0.037, septum 0.76 ± 0.12 vs. 0.83 ± 0.22, *P* = 0.37.

・IL-1β mRNA: peri-infarction area 1.01 ± 0.44 vs. 1.51 ± 0.79, *P* = 0.070, septum 0.51 ± 0.21 vs. 0.71 ± 0.24, *P* = 0.031.

・IL-6 mRNA: peri-infarction area 1.92 ± 1.02 vs. 3.61 ± 1.76, *P* = 0.0092, septum 1.12 ± 1.21 vs. 0.92 ± 0.66, *P* = 0.0092,

**Examination 2**

RT-PCR analysis (Figure 3C)

・SDF1 mRNA: MI model vs. normal; peri-infarction area 2.17 ± 0.48 vs. 0.93 ± 0.16, *P* = 0.0010, septum 1.96 ± 0.96 vs. 1.03 ± 0.25, *P* = 0.064, remote zone; 1.11 ± 0.24 vs. 0.93 ± 0.17; *P* = 0.22

**Examination 3**

Echocardiogram (Figure 5B)

・LVEF: HMGB1 group vs. control; pre 43.00 ± 6.05 vs. 43.92 ± 2.42%, *P* =0.71, 4w later 49.30 ± 3.75 vs. 36.52 ± 3.09%, *P* < 0.0001

RT-PCR analysis (Figure 5C)

・GFP mRNA: peri-infarction area 1.76 ± 0.49 vs. 0.93 ± 0.17, *P* = 0.017

Histological analysis (Figure 5-D2)

・GFP^+^/PDGFRα^+^ cells in the peri-infarction area; 1418.70 ± 243.66 vs. 589.79 ± 66.52/mm^2^, *P* < 0.0001

**Intravital imaging**

Histological analysis (Figure 7-B1)

・GFP^+^/PDGFRα^+^ cells in the peri-infarction area; 1516.5 ± 132.5 vs. 689.9 ± 70.6/mm^2^, *P* < 0.001.
